# Peripheral immune profiling identifies CD8⁺ T_EMRA_ and CD4⁺ T TIGIT⁺ cells as independent prognostic markers for first-line immune checkpoint inhibitors in advanced non-small cell lung cancer

**DOI:** 10.1186/s12931-026-03756-6

**Published:** 2026-06-10

**Authors:** Jieun Park, Juwhan Choi, Seunghun Lee, Chae Rin Kim, Yeonwoo Lee, Jihyun Park, Yulim Lee, Young Kee Shin, Sung Yong Lee

**Affiliations:** 1 Research Institute of Pharmaceutical Science, College of Pharmacy, Seoul National University, Seoul, Republic of Korea; 2 Division of Pulmonary, Allergy, and Critical Care Medicine, Department of Internal Medicine, Korea University Guro Hospital, Korea University College of Medicine, 148 Gurodong-ro, Guro-gu, Seoul, Republic of Korea; 3 Department of Molecular Medicine and Biopharmaceutical Sciences, Graduate School of Convergence Science and Technology, Seoul National University, 1 Gwanak-ro, Gwanak-gu, Seoul, Republic of Korea; 4 Laboratory of Molecular Pathology and Cancer Genomics, College of Pharmacy, Seoul National University, Seoul, Republic of Korea; 5 Future Innovation Research Institute, Seoul National University Siheung Campus, Siheung, Gyeonggi-do Republic of Korea

**Keywords:** Non-small cell lung cancer, Immunotherapy, Prognostic biomarker, T lymphocytes

## Abstract

**Background:**

Immune checkpoint inhibitors (ICIs) have demonstrated substantial clinical benefits in advanced non–small cell lung cancer (NSCLC); however, a subset of patients experience early disease progression despite PD-L1 expression, highlighting the need for biomarkers that better reflect systemic immune competence. We performed comprehensive peripheral immune profiling of patients with advanced NSCLC receiving first-line ICI therapy.

**Methods:**

Blood samples were collected from patients with advanced NSCLC prior to the initiation of first-line ICI-based therapy. A total of 23 lymphocyte subsets and 13 soluble immune-related factors were analyzed, and multivariate models were used to identify independent prognostic biomarkers.

**Results:**

A total of 74 patients with advanced NSCLC who received first-line ICI therapy were included in this study, none of whom harbored *EGFR*,* ALK*,* or ROS1* mutations. In multivariate analyses, elevated CD4⁺ T cell immunoreceptor with Ig and ITIM domains (TIGIT)⁺ frequencies independently predicted shorter progression-free survival (hazard ratio (HR) = 3.74, *p* < 0.001), along with increased CD8⁺ terminally differentiated effector memory T cells re-expressing CD45RA (T_EMRA_) cells (HR = 1.98, *p* = 0.01). Consistent with this finding, patients with CD4⁺ T TIGIT⁺ high group exhibited a higher proportion of non-responders (stable or progressive disease) at best response. Non-responder–to–responder transitions between first and best response assessments were observed in the CD4⁺ T TIGIT⁺ low and CD8⁺ T_EMRA_ low groups (*p* = 0.023 and 0.041, respectively), but not in the corresponding high groups. Cytokine profiling revealed significantly lower granzyme B levels in the CD4⁺ T TIGIT⁺ high group (*p* = 0.023).

**Conclusions:**

Baseline frequencies of peripheral CD4⁺ T TIGIT⁺ and CD8⁺ T_EMRA_ cells were independently associated with survival outcomes in advanced NSCLC patients receiving first-line ICI-based therapy, suggesting that peripheral T cell immunophenotyping at treatment initiation may provide prognostic information beyond conventional biomarkers. Further studies incorporating external validation in independent cohorts, longitudinal immune profiling, and deeper T cell subset characterization are warranted to validate these findings and to elucidate the immune dynamics underlying treatment response and resistance.

**Supplementary Information:**

The online version contains supplementary material available at 10.1186/s12931-026-03756-6.

## Introduction

Immune checkpoint inhibitors (ICIs) have revolutionized the treatment landscape of multiple malignancies by restoring antitumor immunity through blocking inhibitory pathways such as programmed death-1 (PD-1), programmed death ligand 1 (PD-L1), and cytotoxic T-lymphocyte-associated protein (CTLA-4) [1–3]. In non-small cell lung cancer (NSCLC), patients with high PD-L1 expression (tumor proportion score (TPS) ≥ 50%) derive the greatest benefit from ICI monotherapy, which led to the first-line FDA approval of pembrolizumab in 2016 [4]. Comparable efficacy has been confirmed using atezolizumab, an anti-PD-L1 monoclonal antibody, and cemiplimab, an anti-PD-1 monoclonal antibody [5, 6]. In addition, several ICI-based combination regimens, most notably ICI plus platinum-based chemotherapy, have demonstrated superior efficacy over chemotherapy alone independent of PD-L1 expression, and these regimens are recommended as first-line options independent of PD-L1 expression in patients without actionable driver alterations [7, 8].

PD-L1 expression remains the primary biomarker used to guide the use of ICIs in clinical practice. Although ICIs provide clinical benefits to a large proportion of patients, there is a subgroup with an inherently poor prognosis, such as those exhibiting disease progression at the first response evaluation. Currently, reliable biomarkers capable of identifying these patients are lacking. Even among patients with high PD-L1 expression (TPS ≥ 50%), only approximately 20% experience long-term disease control, while up to 20% exhibit early progression, and around 60% develop progression after initial stabilization [9]. This limited predictive performance highlights the need not only for effective post-progression treatment strategies but also for biomarkers that better capture the systemic immune state of patients receiving ICI therapy.

Peripheral blood mononuclear cells (PBMCs) offer a readily accessible window into the host immune milieu and reflect both tumor-driven immune modulation and treatment-induced immune remodeling. Indeed, clonal replacement of tumor-infiltrating T cells by newly recruited peripheral T cell clones has been observed following PD-1 blockade [10], and effective cancer immunotherapy has been shown to require an active systemic immune response that extends beyond the tumor itself [11], supporting the relevance of profiling the peripheral T cell compartment in the context of ICI therapy. Several studies have demonstrated that peripheral lymphocyte subsets, particularly those exhibiting features of exhaustion or immunosenescence, which have varying definitions across studies, are associated with ICI efficacy and survival outcomes [12, 13]. In addition, both the differentiation status and exhaustion phenotypes of circulating T cells and circulating cytokines have emerged as potential indicators of immune competence and therapeutic responsiveness [14, 15]. The abnormal elevation of inflammatory cytokine levels has been associated with unfavorable outcomes in patients with advanced NSCLC treated with ICIs [16, 17]. Despite accumulating evidence linking both cellular and soluble immune factors to ICI efficacy, studies that jointly profile peripheral lymphocyte subsets and circulating cytokines in patients receiving first-line ICI-based therapy for advanced NSCLC remain relatively limited. Further exploration of such integrated peripheral immune profiles may help better capture the systemic immune context underlying ICI efficacy and resistance and support the identification of reliable, minimally invasive biomarkers for improved patient stratification in NSCLC.

In this exploratory study, we conducted comprehensive peripheral immune profiling that integrated PBMC-derived lymphocyte subsets and circulating cytokines obtained at baseline, before the initiation of first-line ICI-based therapy, from patients with advanced NSCLC treated with either ICI monotherapy or ICI plus chemotherapy. To minimize technical bias, batch correction was applied to the flow cytometry data before analysis to ensure robust comparability across samples. Using this approach, we aimed to identify baseline immune parameters associated with clinical outcomes and to evaluate their potential as candidate prognostic biomarkers, ultimately underscoring their potential as systemic immune markers to guide therapeutic stratification in patients with advanced NSCLC receiving first-line immunotherapy.

## Materials and methods

### Study design

This study enrolled patients with advanced NSCLC who received first-line ICI therapy at the Korea University Guro Hospital between 2021 and 2023. Patients were excluded if they were younger than 18 years, had a history of other malignancies, or had undergone surgical or other non-ICI systemic treatments prior to enrollment. This study was reviewed and approved by the Ethics Committee for Clinical Research of the Korea University Guro Hospital (IRB No. 2021GR0409) and conducted in accordance with the principles of the Declaration of Helsinki. All patients provided written informed consent to participate in the study. Progression-free survival (PFS) was defined as the time from the initiation of ICI therapy to the date of radiologically confirmed disease progression or death from any cause, whichever occurred first. Disease progression was assessed according to the Response Evaluation Criteria in Solid Tumors (RECIST) v.1.1 [18]. The data cutoff date for this analysis was September 13, 2025. Responders were defined as complete response (CR) or partial response (PR), and non-responders as those with stable disease (SD) or progressive disease (PD), according to the radiologic assessment at the first response evaluation and at the best response during ICI therapy.

### Sample collection and processing

Peripheral blood samples were collected from each patient into ethylenediaminetetraacetic acid (EDTA) tubes before the initiation of first-line ICI-based therapy. Blood samples were processed within 2 h of collection. Whole blood was first centrifuged at 120 × *g* for 20 min, and the supernatant was further centrifuged at 2,500 × *g* for 15 min to obtain plasma. Plasma was aliquoted and stored at -80 °C until analysis.

The lower layer from the initial centrifugation was diluted 1:1 with phosphate-buffered saline (PBS), and PBMCs were isolated using Lymphosep (Biowest, Bradenton, FL, USA; cat. L0560), according to the manufacturer’s instructions. Isolated PBMCs were resuspended in freezing medium (90% fetal bovine serum and 10% dimethyl sulfoxide), aliquoted into cryovials, and stored in liquid nitrogen.

### Cytokine profiling and quantification

Cytokine concentrations, including interleukin (IL)-2, IL-4, IL-6, IL-10, IL-17 A, tumor necrosis factor (TNF), and interferon (IFN)γ, were quantified in plasma using the BD Cytometric Bead Array (CBA) Human Th1/Th2/Th17 Cytokine Kit (BD Biosciences, San Jose, CA, USA; cat#560484) according to the manufacturer’s instructions. Granzyme A (cat# 560299) and granzyme B (cat#560304), IL-8 (cat#558277), and IL-1β (cat#558279) were measured using a BD CBA Human Soluble Protein Master Buffer Kit (cat#558265) and the corresponding CBA Flex Sets (BD Biosciences). Data were acquired on a BD FACSLyric flow cytometer and analyzed using the Flow Cytometric Analysis Program (FCAP) Array™ Software (BD Biosciences). Analyte concentrations were calculated by four-parameter logistic (4-PL) regression against analyte-specific standard curves. Samples for which no concentration could be calculated from the 4-PL model were assigned a value of zero for downstream analyses.

Plasma concentrations of IL-15 and decorin were quantified using the Human IL-15 DuoSet enzyme-linked immunosorbent assay (ELISA) kit (R&D Systems, Minneapolis, MN, USA; cat#DY247-05) and a Human Decorin DuoSet ELISA kit (R&D Systems; cat#DY143), according to the manufacturer’s instructions. Optical density was measured at 450 nm using a microplate reader (Sunrise™, Tecan Trading AG, Männedorf, Switzerland).

### Flow cytometric immunophenotyping of peripheral T cell subsets

Flow cytometry was used to analyze the regulatory T cells (Tregs), exhausted T lymphocytes, and memory T cell subsets. Cryopreserved PBMCs were rapidly thawed at 37 °C, resuspended in flow cytometry buffer (PBS containing 1% bovine serum albumin), and centrifuged at 200 × g for 5 min. The cells were washed once with buffer and stained for live/dead discrimination using a Zombie Aqua Fixable Viability Kit (BioLegend, San Diego, CA, USA; cat#423102), and incubated with Human TruStain FcX™ (BioLegend; cat#422302) to block Fc receptors. Then, antibody cocktails were prepared and incubated with the cells for 30 min at 4 °C in the dark. The following antibodies were used: anti-human CD3 (cat#300324), anti-CD4 (cat#300514), anti-CD8 (cat#344710), anti-CD45RA (cat#304106), anti-CCR7 (cat#353204), anti-CD25 (cat#302606), anti-CD127 (cat#351310), anti-CTLA-4 (cat#349906), anti-TIM-3 (cat#345022), and anti-TIGIT (cat#372709), and anti-PD-1 (cat#329918) (all from BD Biosciences). After staining, the cells were washed, resuspended in buffer (0.3 mL), and analyzed using a BD LSRFortessa or BD FACSLyric flow cytometer (BD Biosciences).

Batch alignment was performed using the Spectre R package [19] after extracting the T cell populations (Supplementary Fig. 1). Only viable cells were included in the downstream flow cytometric analysis. Tregs were defined as CD4^+^, CD25^high^, and CD127^dim^ cells. T cell differentiation subsets were classified based on CD45RA and CCR7 expression as follows: naïve T cells (T naïve, CD45RA⁺CCR7⁺), central memory T cells (T_CM_, CD45RA⁻CCR7⁺), effector memory T cells (T_EM_, CD45RA⁻CCR7⁻), and terminally differentiated effector memory T cells re-expressing CD45RA (T_EMRA_, CD45RA⁺CCR7⁻).

### Statistical analysis

Categorical variables were analyzed using Fisher’s exact test. Within-patient changes between the first and best responses were evaluated using McNemar’s test for paired categorical data. Survival curves were estimated using the Kaplan–Meier method and compared using the log-rank test. Optimal cutoff values were determined using the MaxStat R package. Univariate and multivariate analyses were performed using Cox proportional hazard regression models. To minimize overfitting and identify the most informative variables, the least absolute shrinkage and selection operator (LASSO) regression was applied for variable selection. As univariate analyses served solely as a screening step with final conclusions drawn from multivariate models, false discovery rate (FDR) correction was not applied. Statistical significance was defined as *p* < 0.05. All statistical analyses and graphical visualizations were performed using R software (v.4.2.3; R Foundation for Statistical Computing, Vienna, Austria) and FlowJo software (v.10.0.7; FlowJo LLC, Ashland, OR, USA).

## Results

### Clinicopathological characteristics of enrolled patients

A total of 74 patients with advanced NSCLC who received first-line ICI therapy were included in this study, none of whom harbored *EGFR*, *ALK*, or *ROS1* mutations. Their baseline characteristics are summarized in Table 1. The median age was 72 years (range, 50–95 years), and most patients were male (*n* = 67, 90.5%). Disease stage was IVA in 33 patients (44.6%) and IVB in 41 patients (55.4%). Most tumors were adenocarcinomas (*n* = 39, 52.7%). Eastern Cooperative Oncology Group (ECOG) performance status was available for 29 patients, of whom 19 (65.5%) had a score of 0–1 and 10 (34.5%) had a score of ≥ 2; status was not recorded for the remaining 45 patients. PD-L1 TPS was ≥ 50% in 29 patients (39.2%) and < 50% in 45 patients (60.8%). Approximately 70% of patients received ICI in combination with chemotherapy, whereas the remaining patients were treated with ICI monotherapy. Based on the best overall response, the objective response rate (ORR) was 43.2%. At the data cutoff point, the median time to censoring for the PFS analysis was 13.1 months. Overall, 63 patients (85.1%) experienced disease progression, and 51 died (68.9%).

**Table 1:** Patient characteristics

	*N* (%)
Age (years)	
Median (min–max)	72 (50–95)
< 70	28 (37.8)
≥ 70	46 (62.2)
Sex	
Male	67 (90.5)
Female	7 (9.5)
Stage	
IVA	33 (44.6)
IVB	41 (55.4)
Histology	
Adenocarcinoma	39 (52.7)
Squamous cell carcinoma	27 (36.5)
Others	8 (10.8)
ECOG performance score	
0, 1	19 (65.5)
≥ 2	10 (34.5)
NA	45
Treatment regimen	
ICI monotherapy	21 (28.4)
ICI plus chemotherapy	53 (71.6)
PD-L1 IHC (clone: SP263)	
< 50	45 (60.8)
≥ 50	29 (39.2)
Best response	
CR	3 (4.1)
PR	29 (39.2)
SD	16 (21.6)
PD	26 (35.1)
DCR, %	64.9%
ORR, %	43.2%
ICI-induced pneumonitis	
No	56 (75.7)
Yes	18 (24.3)
ICI hold due to irAE	
No	69 (93.2)
Yes	5 (6.8)

### Baseline distribution of memory and exhausted T cell subsets according to clinical response

**Fig. 1:**
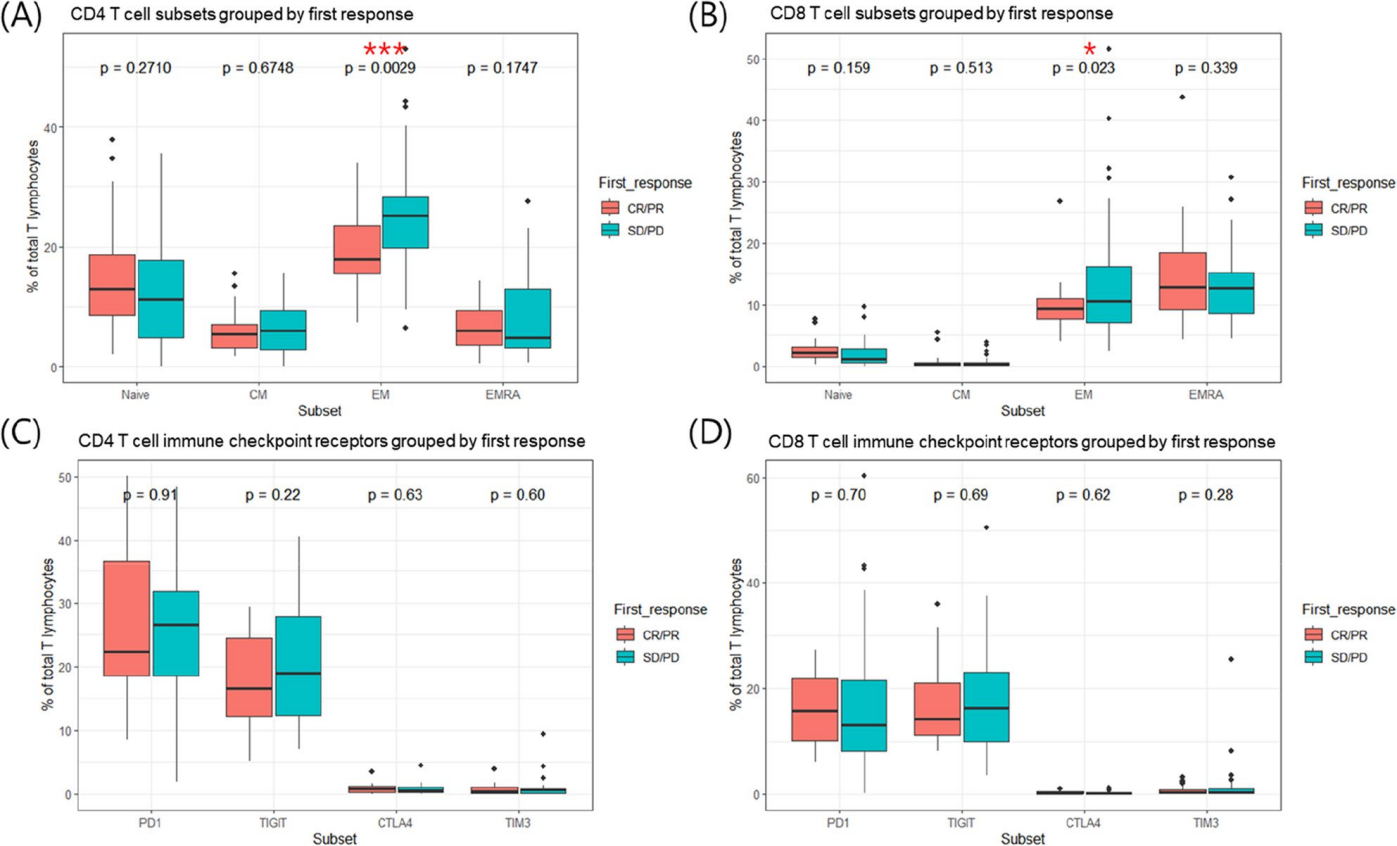
Distinct distribution of memory and exhausted T cell subsets between responders and non-responders. **A**, **B** Box plots showing the proportions of CD4⁺ and CD8⁺ T cells across differentiation stages (Naïve, CM, EM, and T_EMRA_) in patients stratified by first response. **C**, **D** Proportions of cells positive for each immune checkpoint receptors in CD4⁺ and CD8⁺ T cells were compared between responders and non-responders. Each box represents the interquartile range with median values; whiskers indicate 1.5 × interquartile range (IQR(. CM, central memory; EM, effector memory; T_EMRA_, terminally differentiated effector memory T cells re-expressing CD45RA

To characterize the baseline T cell subsets associated with treatment outcomes, we compared the frequencies of CD4^+^ and CD8^+^ T cell memory subsets, including T naïve, T_CM_, T_EM_, and T_EMRA,_ as well as cells expressing immune checkpoint receptors, between responders and non-responders, defined by the first response assessment (CR/PR vs. SD/PD) (Fig. 1). Gating strategy, representative plots, and Uniform Manifold Approximation and Projection (UMAP) visualization before and after batch alignment using Spectre are provided in Supplementary Fig. 1. Responders exhibited a significantly lower proportion of CD4⁺ T_EM_ and CD8⁺ T_EM_ subsets compared with non-responders (*p* = 0.0029 in CD4⁺ T_EM_ and *p* = 0.023 in CD8⁺ T_EM_). In contrast, the proportions of cells positive for each immune checkpoint receptors, including PD-1, TIGIT, CTLA-4, and TIM-3, in CD4^+^ and CD8^+^ T cells was not significantly different between the two groups.

### Identification of independent prognostic biomarkers

To identify independent prognostic biomarkers in patients with advanced NSCLC receiving first-line ICI therapy, we first assessed whether major clinical variables were associated with PFS. Kaplan-Meier analyses showed no significant differences in PFS according to treatment regimen combined with PD-L1 TPS (p = 0.56) or histologic subtype (p = 0.82; Supplementary Fig. 2). We then analyzed 13 soluble factors and 23 lymphocyte subsets obtained from peripheral blood. Decorin, a secreted matrix-associated protein with immunomodulatory potential, was analyzed together with the soluble factors. For each continuous variable, patients were classified as ‘high’ if the value was above the cutoff and ‘low’ if at or below. Cutoff values and univariate analysis results for all variables are summarized in Supplementary Table 3. In univariate analyses, PD-L1 immunohistochemistry (IHC( expression and age at diagnosis showed hazard ratios (HRs) of 0.82 (95% confidence interval (CI) 0.49–1.37, *p* = 0.46) and 1.2 (95% CI 0.71–2.0, *p* = 0.53), respectively, for PFS.

Two models were constructed to identify independent prognostic biomarkers. In both models, disease stage (IVA vs. IVB) and treatment regimen (ICI monotherapy vs. ICI plus chemotherapy) were included as predefined clinical covariates. Model 1 included variables with *p* < 0.1 in both PFS and overall survival (OS) in univariate analyses, whereas Model 2 applied LASSO regression to select six variables based on the number of PFS events before multivariate testing (Table 2).

**Table 2:** Univariate and multivariate analysis

	Univariate analysis				Multivariate analysis			
	PFS		OS		PFS		OS	
	Hazard ratio (95% CI)	*p*-value	Hazard ratio (95% CI)	*p*-value	Hazard ratio (95% CI)	*p*-value	Hazard ratio (95% CI)	*p*-value
Model 1. Univariate analysis (*p* < 0.1 in both PFS and OS)								
Stage (IVA vs. IVB)	0.78 (0.47–1.3)	0.34	1.3 (0.75–2.3)	0.33	0.79 (0.45–1.38)	0.403	1.61 (0.83–3.1)	0.159
Treatment	1.36 (0.77–2.41)	0.29	1.0 (0.55–1.8)	0.99	0.98 (0.54–1.78)	0.95	0.92 (0.47–1.8)	0.8
IL-6	2.7 (0.94–7.5)	0.064	4.4 (1.1–18)	0.043	1.85 (0.62–5.52)	0.269	2.09 (0.45–9.8)	0.349
PERLS	0.4 (0.18–0.87)	0.021	0.42 (0.19–0.95)	0.036	0.56 (0.23–1.34)	0.191	0.46 (0.16–1.3)	0.139
Treg PD-1^+^	0.52 (0.29–0.93)	0.027	0.52 (0.27-1)	0.057	0.54 (0.30–0.99)	0.045 (*)	0.58 (0.28–1.2)	0.134
CD4^+^T TIGIT^+^	2.9 (1.5–5.7)	0.0021	2.2 (1.1–4.3)	0.025	2.77 (1.34–5.69)	0.006 (**)	1.84 (0.82–4.1)	0.142
CD4^+^ T_EM_	3 (1.1–8.2)	0.038	2.9 (0.9–9.4)	0.076	2.45 (0.86–6.99)	0.093	2.93 (0.87–9.8)	0.082
CD8^+^ T_EMRA_	1.8 (1.1-3)	0.02	1.7 (0.95–2.9)	0.074	2.05 (1.20–3.50)	0.009 (**)	2.05 (1.08–3.9)	0.028 (*)
Model 2. Six variables selected by LASSO (PFS-based)								
Stage (IVA vs. IVB)	-	-	-	-	0.92 (0.54–1.6)	0.766	1.42 (0.76–2.7)	0.274
Treatment	-	-	-	-	1.35 (0.71–2.5)	0.357	1.00 (0.51-2.0)	0.993
Treg PD-1^+^	-	-	-	-	0.61 (0.33–1.1)	0.116	0.50 (0.24-1.0)	0.059
CD4^+^T TIGIT^+^	-	-	-	-	3.06 (1.42–6.6)	0.004 (***)	2.85 (1.33–6.1)	0.007 (**)
CD8^+^T CTLA4^+^	0.47 (0.27–0.8)	0.0059	0.9 (0.5–1.6)	0.74	0.79 (0.43–1.5)	0.455	1.27 (0.60–2.7)	0.536
CD8^+^ T Naïve	0.54 (0.32–0.89)	0.016	0.85 (0.48–1.5)	0.56	0.67 (0.36–1.3)	0.215	1.07 (0.54–2.1)	0.854
CD8^+^ T_EMRA_	-	-	-	-	2.03 (1.16–3.6)	0.013 (*)	2.09 (1.08-4.0)	0.028 (*)
CD8^+^ T_CM_	0.52 (0.31–0.87)	0.012	0.74 (0.43–1.3)	0.29	0.61 (0.33–1.1)	0.113	0.65 (0.33–1.3)	0.227

Dashed line (–) indicates duplicate data

*PFS *progression-free survival, *OS *overall survival, *PERLS *CD8^+^ T cell PD-1^+^ to CD4^+^ T cell PD-1^+^ ratio, *EM *effector memory, *T*_*EMRA*_ terminally differentiated effector memory T cells re-expressing CD45RA, *CM *central memory, *CI *confidence interval

In Model 1, multivariate analysis revealed that CD4⁺ T TIGIT⁺ (TIGIT-expressing CD4^+^ T cell; HR 2.77, 95% CI 1.34–5.69, *p* = 0.006) and CD8⁺ T_EMRA_ (HR 2.05, 95% CI 1.20–3.50, *p* = 0.009) were independent prognostic factors for PFS. The PD-1⁺ Treg (PD-1-expressing regulatory T cells; HR 0.54, 95% CI 0.30–0.99, *p* = 0.045) also remained significantly associated, whereas CD4⁺ T_EM_ was marginally associated (HR 2.45, 95% CI 0.86–6.99, *p* = 0.093). In Model 2, only CD4⁺ T TIGIT⁺ (HR 3.06, 95% CI 1.42–6.60, *p* = 0.004) and CD8⁺ T_EMRA_ (HR 2.03, 95% CI 1.16–3.60, *p* = 0.013) remained statistically significant independent predictors for PFS. For overall survival, CD8⁺ T_EMRA_ was independently associated with prognosis in both Model 1 (HR 2.05, 95% CI 1.08–3.90, *p* = 0.028) and Model 2 (HR 2.09, 95% CI 1.08–4.00, *p* = 0.028), and CD4⁺ T TIGIT⁺ was independently associated with OS in Model 2 (HR 2.85, 95% CI 1.33–6.10, *p* = 0.007).

**Fig. 2:**
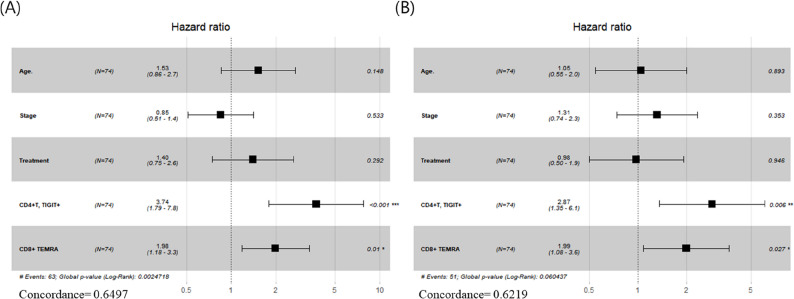
Multivariable Cox proportional hazards analysis for (**A**) progression-free survival and (**B**) overall survival. Forest plots show hazard ratios (HR) with 95% confidence intervals. The model included age(≥70yrs), stage (IVA vs. IVB), treatment modality (ICI mono vs. ICI plus chemotherapy), peripheral CD4⁺ T TIGIT⁺ (Low vs. High), and CD8⁺ T_EMRA_ (Low vs. High). Squares and horizontal lines represent HR and 95% CI, respectively. ICI, immune checkpoint inhibitor

To further assess the independent prognostic value of the two biomarkers consistently selected across Model 1 and Model 2, a reduced multivariable Cox regression model was constructed using only CD4⁺ T TIGIT⁺ and CD8⁺ T_EMRA_ together with the clinical covariates age (≥ 70 years), disease stage (IVA vs. IVB), and treatment regimen (ICI monotherapy vs. ICI plus chemotherapy) (Fig. 2). In this model, both CD4⁺ T TIGIT⁺ (HR 3.74, 95% CI 1.79–7.80, *p* < 0.001) and CD8⁺ T_EMRA_ (HR 1.98, 95% CI 1.18–3.30, *p* = 0.01) remained independent prognostic factors for PFS. Similar results were observed for OS (CD4⁺ T TIGIT⁺: HR 2.87, 95% CI 1.35–6.10, *p* = 0.006; CD8⁺ T_EMRA_: HR 1.99, 95% CI 1.08–3.60, *p* = 0.027). The concordance index was 0.65 for the PFS model and 0.62 for the OS model. Bootstrap-validated hazard ratios (B = 200 resamples) for all three multivariable models — Table 2 Model 1, Table 2 Model 2, and the final reduced model shown in Fig. 2 — are provided in Supplementary Table 4. The bootstrap estimates for CD4⁺ T TIGIT⁺ and CD8⁺ T_EMRA_ were directionally consistent with the original point estimates across all three models, with 95% CIs excluding the null value of 1.

### Prognostic impact of CD4⁺ T TIGIT⁺ and CD8⁺ T_EMRA_ subsets on survival

**Fig. 3:**
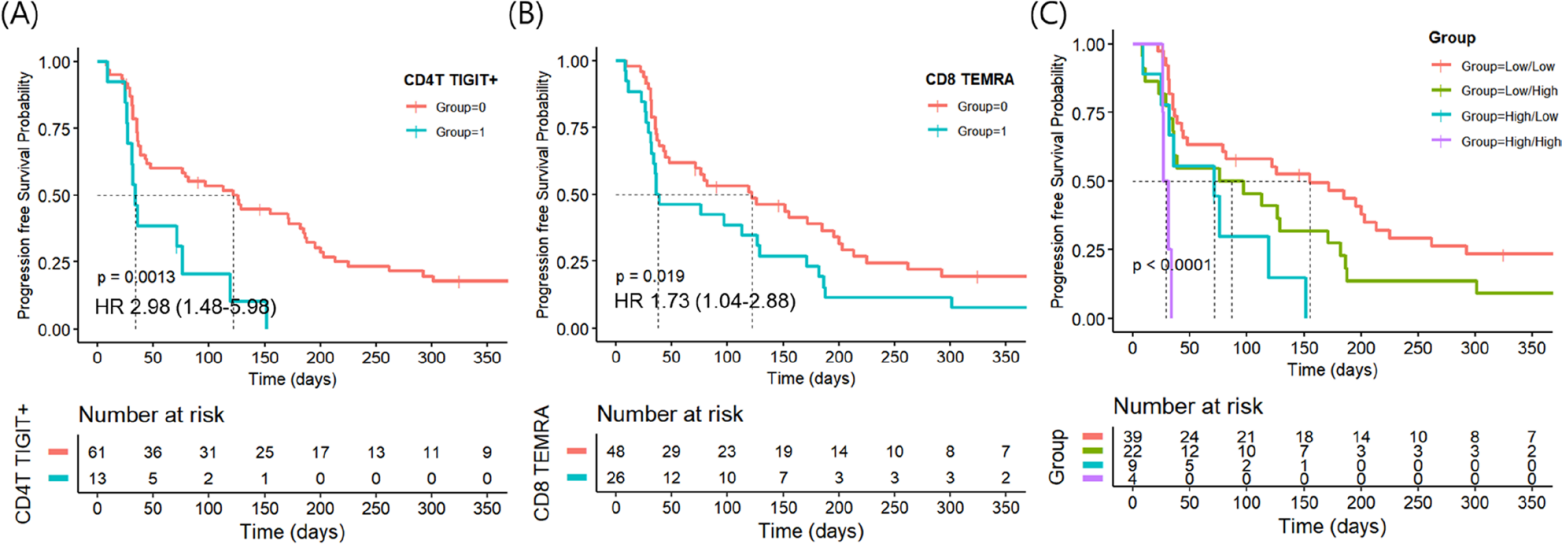
Prognostic impact of CD4⁺ T TIGIT⁺ and CD8⁺ T_EMRA_ subsets on PFS. Kaplan–Meier curves show PFS differences according to (**A**) CD4⁺ T TIGIT⁺ level (low vs. high), (**B**) CD8⁺ T_EMRA_ level (low vs. high), and (**C**) combined CD4⁺ T TIGIT⁺/CD8⁺ T_EMRA_ subgroups (low/low, low/high, high/low, high/high). Statistical significance was assessed using the log-rank test. Hazard ratio (HR) was estimated by Cox proportional hazards model stratified by treatment modality (ICI monotherapy vs. ICI plus chemotherapy)

Based on these results, Kaplan–Meier analyses were performed using CD4⁺ T TIGIT⁺ and CD8⁺ T_EMRA_ to further evaluate their prognostic impact on survival outcomes (Fig. 3). Both CD4⁺ T TIGIT⁺ and CD8⁺ T_EMRA_ were significantly associated with poor prognosis (*p* = 0.0013 and *p* = 0.019, respectively; treatment-stratified Cox HR 2.88 (95% CI 1.47–5.67) and 1.82 (95% CI 1.10–3.03)) (Fig. 3A and B). Patients were then stratified into four groups according to the combined status of the two parameters, defined as CD4⁺ T TIGIT⁺ (low vs. high) and CD8⁺ T_EMRA_ (low vs. high), and survival analysis revealed a graded prognostic pattern (Fig. 3C). Compared with the reference group in which both markers were low (CD4⁺ T TIGIT⁺ low / CD8⁺ T_EMRA_ low), patients with CD4⁺ T TIGIT⁺ low / CD8⁺ T_EMRA_ high (treatment-strafied Cox HR 1.79, 95% CI 1.02–3.15), CD4⁺ T TIGIT⁺ high / CD8⁺ T_EMRA_ low (treatment-strafied Cox HR 3.01, 95% CI 1.30–6.97), and CD4⁺ T TIGIT⁺ high / CD8⁺ T_EMRA_ high (treatment-strafied Cox HR 8.62, 95% CI 2.61–28.41) showed progressively poorer PFS, with statistically significant differences (*p* < 0.0001).

When stratified by treatment modality (Supplementary Fig. 5), no significant difference in PFS was observed according to CD4⁺ T TIGIT⁺ status in the ICI monotherapy cohort (*p* = 0.47; HR 2.6, 95% CI 0.95–7.2); however, interpretation was limited by the very small number of patients in the CD4⁺ T TIGIT⁺-high group (*n* = 1). In contrast, in patients treated with ICI plus chemotherapy, elevated CD4⁺ T TIGIT⁺ cells were associated with significantly poorer PFS (HR 3.1, 95% CI 1.5–6.5, *p* = 0.0019), with a consistent directional effect across both regimens despite the limited statistical power in the monotherapy subgroup. Consistently, analysis of plasma cytokine profiling demonstrated significantly lower granzyme B concentrations in the CD4⁺ T TIGIT⁺ high group compared with the low group (*p* = 0.023) (Supplementary Fig. 5C).

**Fig. 4:**
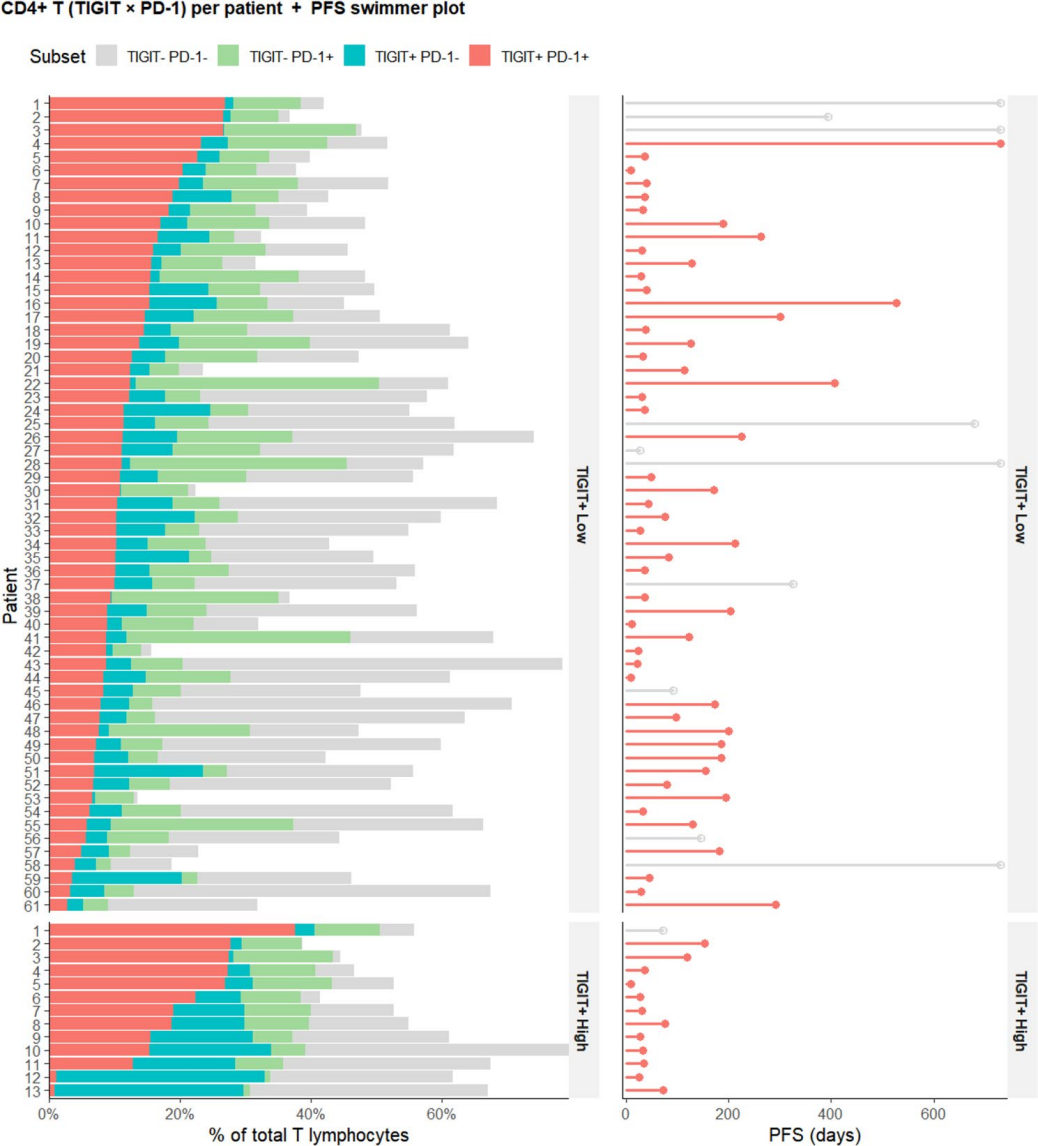
CD4⁺ T-cell TIGIT and PD-1 co-expression subset distribution per patient and corresponding progression-free survival (PFS) swimmer plot. The left panel illustrates the per-patient distribution of TIGIT and PD-1 co-expression subsets within CD4⁺ T cells. The right panel shows the corresponding PFS in a swimmer-plot format, where red bars denote patients who experienced a progression event and gray bars indicate patients without an event at the time of data cutoff

To further characterize the phenotypic heterogeneity of CD4⁺ T TIGIT⁺ cells at the individual patient level, the distribution of TIGIT and PD-1 co-expression subsets was visualized per patient, together with corresponding PFS using a swimmer plot (Fig. 4). CD4⁺ T TIGIT⁺ cells predominantly co-expressed PD-1 across most patients; however, a subset of patients exhibited a substantial proportion of TIGIT⁺PD-1⁻ CD4⁺ T cells. Patient-level Spearman correlation analysis further indicated that the frequency of CD4⁺ T TIGIT⁺ cells was positively correlated with CD4⁺ T_EM_ (*r* = 0.244, *p* = 0.036) and CD4⁺ T_EMRA_ (*r* = 0.244, *p* = 0.036), but not with CD4⁺ Treg (*r* = 0.149, *p* = 0.21) or CD8⁺ T_EMRA_ (*r* = − 0.192, *p* = 0.10) (Supplementary Fig. 6), suggesting an effector/memory-associated rather than regulatory phenotype.

### Association of CD4⁺ T TIGIT⁺ and CD8⁺ T_EMRA_ subsets with treatment response

We next examined the association between the two independent prognostic factors—CD4⁺ T TIGIT⁺ and CD8⁺ T_EMRA_—and treatment response. Both markers were analyzed according to their low and high groups in relation to the first and best response.

**Fig. 5:**
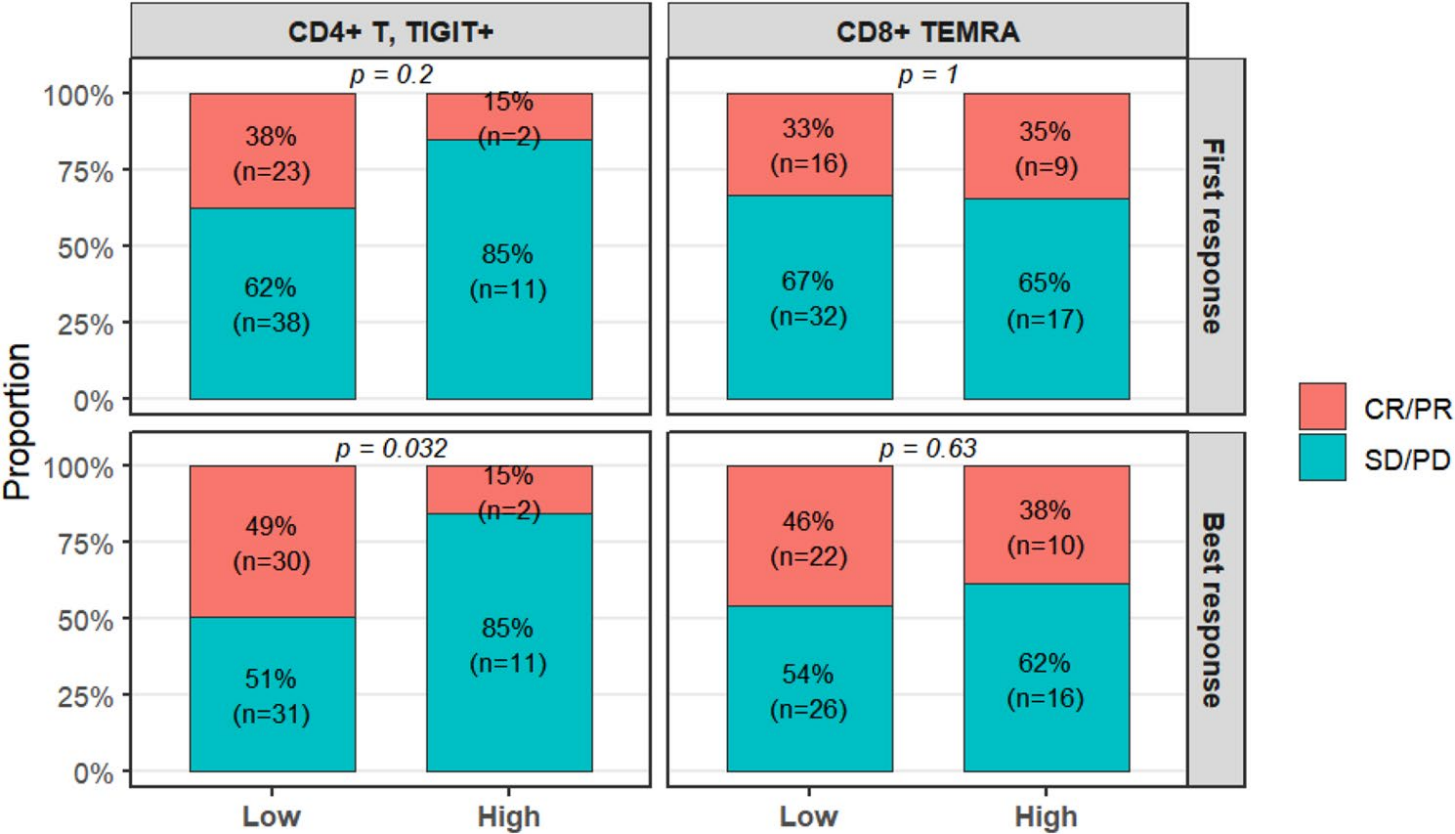
Association of CD4^+^ T TIGIT^+^ and CD8^+^ T_EMRA_ with first response and best response. For each continuous variable, an optimal cutoff value was determined using the MaxStat R package, and patients were classified as 'high' if the value was above the cutoff and 'low' if at or below. CM, central memory; EM, effector memory; T_EMRA_, terminally differentiated effector memory T cells re-expressing CD45RA; CR/PR, complete response/partial response; SD/PD, stable disease/progressive disease

The CD4⁺ T TIGIT⁺ high group showed a higher proportion of non-responders than the CD4⁺ T TIGIT⁺ low group, with a statistically significant difference observed for the best response (*p* = 0.032) (Fig. 5). In contrast, the proportion of responders did not differ significantly between the CD8^+^ T_EMRA_ low and high groups for either the first or best response (*p* = 1.0 and *p* = 0.63, respectively).

Changes in treatment response from the first to the best assessment were further evaluated within each biomarker subgroup using McNemar’s test. A significant change in response category was observed in the CD4⁺ T TIGIT⁺ low group (*p* = 0.023), but not in the CD4⁺ T TIGIT⁺ high group (*p* = 1.0). A similar pattern was observed for CD8 + T_EMRA_, with a significant change in the low group (*p* = 0.041), but not in the high group (*p* = 1.0). Consistently, conversion from non-responders to responders was more frequent in the CD4⁺ T TIGIT⁺ low group (18.4%, *n* = 7) than in the CD4⁺ T TIGIT⁺ high group (0%, *n* = 0), and in the CD8⁺ T_EMRA_ low group (18.7%, *n* = 6) than in the CD8⁺ T_EMRA_ high group (5.9%, *n* = 1).

## Discussion

In this study, we comprehensively profiled peripheral immune parameters, including PBMC-derived lymphocyte subsets and circulating immune-related factors in patients with advanced NSCLC receiving first-line ICI-based therapy. Among the immune parameters evaluated, baseline CD4⁺ T TIGIT⁺ and CD8⁺ T T_EMRA_ subsets emerged as the most robust and consistent prognostic biomarkers, each independently associated with shorter PFS in multivariable analyses.

Current approved tissue-based biomarkers for first-line ICI selection in NSCLC have well-recognized limitations. PD-L1 expression, the principal biomarker in routine practice, is constrained by spatial and temporal heterogeneity and by assay variability across platforms [20, 21], and tumor-agnostic biomarkers such as tumor mutation burden (TMB) and microsatellite instability-high (MSI-H) have restricted applicability in this disease given the rarity of MSI-H and the absence of a universally accepted TMB threshold [22]. These limitations motivated the research for complementary, minimally invasive biomarkers.

Peripheral blood immune profiling offers an accessible readout of systemic immune status that is mechanistically engaged by PD-1 blockade. Recent work has shown that the expanded tumor-reactive T cell clones observed after anti-PD-1 therapy are not predominantly derived from pre-existing tumor-infiltrating lymphocytes, but rather from novel clonotypes recruited from peripheral sources [10]. This indicates that the systemic T cell pool is essential for an effective tumor immune response. Consistently, early proliferative responses of circulating PD-1⁺ CD8⁺ T cells have been linked to clinical benefit [23], and peripheral CD4⁺ T cell immunity has emerged as a critical determinant of response to PD-1 blockade in NSCLC [24]. Our identification of baseline CD4⁺ T TIGIT⁺ and CD8⁺ T_EMRA_ subsets as independent prognostic markers is consistent with, and extends, this body of evidence by highlighting specific circulating subsets relevant to first-line ICI outcomes.

Flow cytometry–based immunophenotyping of peripheral lymphocytes is inherently susceptible to batch effects across experimental runs, which can compromise reproducibility and cross-sample comparability [25]. Although standardized acquisition and gating protocols can reduce such effects, they cannot eliminate them, and manual gating adjustments remain inherently subjective [26]. To overcome these limitations, various computational tools have been developed to correct batch-dependent variations and enable the reliable integration of multi-batch cytometry data [27, 28]. We employed the Spectre R package to perform quantitative batch alignment across our flow cytometry datasets, harmonizing distributions between batches while preserving biological variation [19]. This approach enhanced analytical robustness and supported the identification of prognostic immune determinants in a multi-batch cohort.

At the first response assessment, both CD4^+^ and CD8^+^ T_EM_ cells were significantly enriched in non-responders. This finding is consistent with a recent report demonstrating that the accumulation of highly differentiated CD8^+^ T_EM_ cells in peripheral blood is associated with impaired proliferative capacity and poor responsiveness to ICI therapy in patients with NSCLC [29]. In contrast, immune checkpoint receptors, including TIGIT, did not significantly differ between responders and non-responders at this early time point, even though baseline CD4⁺ T TIGIT⁺ frequency subsequently emerged as an independent prognostic factor for PFS. This apparent discrepancy is not unexpected, as early response and PFS capture related but distinct endpoints: the former reflects tumor-intrinsic sensitivity to treatment as a binary outcome, whereas PFS integrates the durability of disease control over time. Moreover, dichotomized cutoff-based analyses identify patients at the extreme of the distribution, in whom the prognostic signal is concentrated.

The peripheral CD4⁺ T TIGIT⁺ high group observed in prognostically unfavorable patients likely reflects a systemic immune status skewed toward inhibitory and regulatory signaling rather than effective antitumor activation. TIGIT, an inhibitory immune checkpoint receptor, plays a critical role in dampening antitumor immunity by suppressing T cell activation and promoting Treg-mediated immune tolerance [30]. Consistent with this, previous studies have demonstrated that high TIGIT expression within the tumor microenvironment correlates with poor prognosis and reduced responsiveness to PD-(L)1 blockade [31, 32]. Moreover, CD4⁺ T TIGIT⁺ cells have been reported to exhibit diminished effector activity while fostering an immunoregulatory environment that supports tumor persistence [33]. Although these observations were primarily from tumor tissues, our findings in peripheral blood suggest that enrichment of circulating CD4⁺ T TIGIT⁺ cells may capture an immunosuppressive phenotype at the systemic level. Supporting this interpretation, patients with high CD4⁺ T TIGIT⁺ group also exhibited significantly lower plasma granzyme B levels, consistent with reduced systemic cytotoxic activity. Notably, the unfavorable prognostic impact of CD4⁺ T TIGIT⁺ enrichment persisted in patients treated with ICI combined with chemotherapy, indicating that this immune phenotype may not be fully overcome by the addition of cytotoxic chemotherapy to PD-(L)1 blockade. Furthermore, significant shifts from non-responder to responder status between first and best response assessments were observed only in patients with CD4⁺ T TIGIT⁺-low group, whereas no such transitions occurred in the CD4⁺ T TIGIT⁺-high group, suggesting an association between a baseline inhibitory-receptor–dominant peripheral state and reduced likelihood of response deepening, although causal inference is not possible from this cross-sectional analysis. We note, however, that TIGIT is expressed across multiple CD4⁺ subsets, including Tregs; further phenotypic and functional characterization will be needed to define the biology of the specific subset captured by this marker. Of note, patient-level correlation analyses in our cohort revealed modest but significant positive correlations between CD4⁺ T TIGIT⁺ frequency and both CD4⁺ T_EM_ and CD4⁺ T_EMRA_ subsets, but not with Treg frequency, suggesting that the prognostic CD4⁺ T TIGIT⁺ cells in our cohort reflect enrichment within the differentiated effector-memory compartment rather than a Treg-dominated population. Nevertheless, as these observations were derived from baseline peripheral immune profiling and associative analyses, they should be interpreted with caution. Further studies are warranted to elucidate the underlying biological mechanisms and determine whether this immune state can be therapeutically modulated.

Baseline CD8⁺ T_EMRA_ high group was likewise independently associated with shorter PFS in our cohort. T_EMRA_ cells represent a terminally differentiated effector population characterized by short telomeres, frequent loss of CD27 and CD28 co-stimulatory receptors, and acquisition of a senescence-associated phenotype, features that have been linked to reduced proliferative capacity and impaired responsiveness to TCR-driven activation [29, 34, 35]. Consistent with this, peripheral expansion of highly differentiated or senescent CD8⁺ T cells has been associated with poor clinical responses to ICI therapy in lung cancer across multiple independent cohorts [13, 29]. However, the biology of circulating T_EMRA_ cells in the context of ICI is not uniformly unfavorable: within the T_EMRA_ compartment, CX3CR1⁺ effector-like subsets have been reported to expand early during anti-PD-1 therapy in responders and to associate with favorable outcomes in NSCLC [36], suggesting that the net prognostic signal from bulk T_EMRA_ frequencies may reflect the balance between senescent and functionally competent effector subpopulations [37]. In our cohort, as with CD4⁺ T TIGIT⁺, non-responder-to-responder transitions at best response were observed at a significantly higher rate in patients with CD8⁺ T_EMRA_ low group, consistent with an association between a less terminally differentiated baseline peripheral T-cell pool and a higher likelihood of response deepening over time, though longitudinal profiling will be required to establish any directional relationship. Further subset-level phenotyping and functional experiments will be required.

In contrast to the cellular findings, parallel analyses of 13 circulating immune-related factors examined in this study showed limited independent prognostic value. In univariate Cox analysis, four pro-inflammatory mediators, such as IFNγ, IL-6, IL-4, and IL-8, showed a marginally associated with PFS (*p* < 0.1) with uniformly elevated hazard ratios (HR ≥ 1.5), consistent with a directional trend linking higher circulating inflammation to unfavorable outcomes. However, none was selected by LASSO regularization, suggesting that their prognostic information overlapped with, or was weaker than, that carried by the cellular parameters. Although inflammatory mediators such as IL-6, IL-8, and TNF have been implicated in modulating the immune milieu [38–40], soluble factors are subject to substantial inter- and intra-individual variability and sensitivity to sampling conditions, and a single pretreatment measurement may not adequately capture their dynamic contribution during ICI therapy.

This study had several limitations. The sample size was modest and derived from a single cohort, and cutoff values for immune subsets were determined using maximally selected rank statistics, which may increase the risk of overfitting and limit the generalizability of our findings, underscoring the need for validation in larger, independent cohorts. Patients receiving ICI monotherapy and those treated with ICI combined with chemotherapy were analyzed together due to limited sample size; although no significant PFS difference was observed between treatment groups, regimen-specific effects cannot be excluded. In addition, functional assays were not performed to directly confirm T cell exhaustion states, and peripheral immune parameters, while informative of systemic immune status, do not directly recapitulate the tumor microenvironment. Finally, all samples were obtained at baseline; accordingly, our findings describe prognostic associations with pretreatment peripheral immune states and do not allow inference regarding treatment-induced immune dynamics over time.

## Conclusions

In conclusion, the present study identified that elevated baseline peripheral CD4⁺ T TIGIT⁺ and CD8⁺ T_EMRA_ cells are independent prognostic biomarkers for shorter PFS in patients with advanced NSCLC receiving first-line ICI. These findings suggest that a systemically immunosenescent or exhaustion-skewed peripheral T cell phenotype at treatment initiation is associated with inferior outcomes, which underscores the potential value of baseline peripheral immune profiling as a complement to existing biomarkers such as PD-L1 expression. Further studies with external validation in independent cohorts, serial monitoring, and deeper T cell subset characterization essential to confirm the clinical utility of these biomarkers. 

## Supplementary Information

Supplementary Material 1.

## Data Availability

The data that support the findings of this study are available from the corresponding author upon reasonable request.

## Abbreviations

CBA: Cytometric Bead Array
CI: Confidence Interval
CR: Complete Response
CTLA-4: Cytotoxic T-lymphocyte-associated Protein
EDTA: Ethylenediaminetetraacetic Acid
ELISA: Enzyme-linked Immunosorbent Assay
HRs: Hazard Ratios
ICIs: Immune Checkpoint Inhibitors
IFN: Interferon
IHC: Immunohistochemistry
IL: Interleukin
LASSO: Least Absolute Shrinkage and Selection Operator
NSCLC: Non-Small Cell Lung Cancer
OS: Overall Survival
PBMCs: Peripheral Blood Mononuclear Cells
PBS: Phosphate-Buffered Saline
PD: Progressive Disease
PD-1: Programmed Death-1
PD-L1: Programmed Death Ligand 1
PFS: Progression-Free Survival
PR: Partial Response
RECIST: Response Evaluation Criteria in Solid Tumors
SD: Stable Disease
T_EMRA_: Terminally differentiated effector memory T cells re-expressing CD45RA
TIGIT: T cell Immunoreceptor with Ig and ITIM domains
TNF: Tumor Necrosis Factor
TPS: Tumor Proportion Score
Treg: Regulatory T cells
