# Supplementary material for: Peripheral immune profiling identifies CD8⁺ TEMRA and CD4⁺ T TIGIT⁺ cells as independent prognostic markers for first-line immune checkpoint inhibitors in advanced non-small cell lung cancer
Source: Respir Res. 2026 Jun 10;27:309. doi: 10.1186/s12931-026-03756-6 (PMC13479815; doi:10.1186/s12931-026-03756-6)
Supplement: Supplementary file 1 [file 12931_2026_3756_MOESM1_ESM.docx]

**Supplementary Figure 1. Gating strategy for peripheral T cell subsets and inhibitory receptors.** (A) Sequential gating strategy in FlowJo for isolation of CD3+ T cells from PBMCs, including single cell, lymphocyte, viability, and CD3+ gates. (B) UMAP visualization of CD3+ T cells before (left) and after (right) batch alignment using Spectre. Colors indicate individual measurement batches. (C) Representative gating for T cell subset identification after Spectre processing. CD4+ and CD8+ T cells were distinguished by CD4/CD8 expression (left). Memory subsets were defined by CD45RA and CCR7 co-expression, including T naïve, T_CM_, T_EM_, and T_EMRA_ (middle). Tregs were identified as CD4+CD25^high^CD127^low^ cells (right). *PBMC, peripheral blood mononuclear cell; UMAP, Uniform Manifold Approximation and Projection; T_CM_, central memory T cell; T_EM_, effector memory T cell; T_EMRA_, terminally differentiated effector memory T cell re-expressing CD45RA; Treg, regulatory T cell*

**Supplementary Figure 2. Progression-free survival according to treatment regimen combined with PD-L1 TPS and histologic subtype.** Kaplan-Meier curves of progression-free survival stratified by (A) treatment regimen combined with PD-L1 TPS (ICI monotherapy with TPS ≥50%*, ICI plus chemotherapy with TPS <50%, and ICI plus chemotherapy with TPS ≥50%) and (B) histologic subtype. P-values were calculated using the log-rank test.

** In Republic of Korea, ICI monotherapy is not reimbursed for first-line treatment of advanced NSCLC with PD-L1 TPS <50%; this subgroup therefore does not exist in our cohort.*

*TPS, tumor proportion score; ICI, immunotherapy; ADC, adenocarcinoma; SqCC, squamous cell carcinoma*

**Supplementary Table 3. Univariate Cox regression analysis for progression-free survival and cut-off values of all analytes included in the study.**

| Factor | Cutoff | Unit | HR (95% CI) | p-value |
| --- | --- | --- | --- | --- |
| PD-L1 IHC (SP263) | 50 | TPS | 0.82 (0.49-1.37) | 0.46 |
| Age at diagnosis | 70 | yrs | 1.2 (0.71-2) | 0.53 |
| ECOG performance score | 2 |  | 1.2 (0.53-2.8) | 0.65 |
| Stage (IVA vs. IVB) |  |  | 0.78 (0.47-1.3) | 0.34 |
| Treatment (ICI vs. ICI+ChT) |  |  | 1.36 (0.77-2.41) | 0.29 |
| IL-17A | 21.6 | pg/mL | 0.56 (0.24-1.3) | 0.17 |
| IFNr | 2.1 | pg/mL | 1.5 (0.93-2.6) | 0.096 |
| TNF | 1.3 | pg/mL | 1.5 (0.88-2.5) | 0.14 |
| IL-10 | 0 | pg/mL | 1.4 (0.73-2.9) | 0.29 |
| IL-6 | 3.2 | pg/mL | 2.7 (0.94-7.5) | 0.064 |
| IL-4 | 1.8 | pg/mL | 1.6 (0.98-2.7) | 0.062 |
| IL-2 | 0 | pg/mL | 1.5 (0.9-2.5) | 0.12 |
| IL-15 | 3.5 | pg/mL | 1.5 (0.78-2.8) | 0.23 |
| IL-1β | 0 | pg/mL | 1.2 (0.64-2.5) | 0.52 |
| IL-8 | 7.2 | pg/mL | 1.8 (0.97-3.3) | 0.062 |
| Decorin | 8390.9 | pg/mL | 0.63 (0.29-1.4) | 0.25 |
| Granzyme A | 10 | pg/mL | 2.1 (1-4.5) | 0.051 |
| Granzyme B | 13.2 | pg/mL | 0.71 (0.43-1.2) | 0.18 |
| CD8+ T/CD4+ T ratio | 0.3 |  | 0.42 (0.17-1) | 0.052 |
| PERLS | 0.2 |  | 0.4 (0.18-0.87) | 0.021 |
| CD4^+^ T | 60.9 | % of total T lymphocytes | 1.7 (0.99-3.1) | 0.056 |
| CD8^+^ T | 14.8 | % of total T lymphocytes | 0.41 (0.18-0.92) | 0.03 |
| Double negative T | 21.5 | % of total T lymphocytes | 0.46 (0.27-0.77) | 0.0029 |
| CD4^+^ Treg | 2.77 | % of total T lymphocytes | 0.58 (0.35-0.97) | 0.036 |
| CD4^+^ Treg, PD1^+^ | 2.73 | % of total T lymphocytes | 0.52 (0.29-0.93) | 0.027 |
| CD4^+^ T, PD1^+^ | 36.69 | % of total T lymphocytes | 0.43 (0.2-0.94) | 0.034 |
| CD4^+^ T, CTLA4^+^ | 0.61 | % of total T lymphocytes | 0.62 (0.37-1) | 0.061 |
| CD4^+^ T, TIGIT^+^ | 28.22 | % of total T lymphocytes | 2.9 (1.5-5.7) | 0.0021 |
| CD4^+^ T, TIM3^+^ | 1.07 | % of total T lymphocytes | 0.53 (0.25-1.1) | 0.097 |
| CD8^+^ T, PD1^+^ | 6.76 | % of total T lymphocytes | 0.39 (0.19-0.77) | 0.0065 |
| CD8^+^ T, CTLA4^+^ | 0.11 | % of total T lymphocytes | 0.47 (0.27-0.8) | 0.0059 |
| CD8^+^ T, TIGIT^+^ | 26.62 | % of total T lymphocytes | 2 (0.99-3.9) | 0.052 |
| CD8^+^ T, TIM3^+^ | 1.85 | % of total T lymphocytes | 0.45 (0.19-1.1) | 0.07 |
| CD4^+^ T Naïve | 11.13 | % of total T lymphocytes | 0.65 (0.39-1.1) | 0.094 |
| CD4^+^ T_CM_ | 2.89 | % of total T lymphocytes | 0.64 (0.37-1.1) | 0.11 |
| CD4^+^ T_EM_ | 10.83 | % of total T lymphocytes | 3 (1.1-8.2) | 0.038 |
| CD4^+^ T_EMRA_ | 1.22 | % of total T lymphocytes | 2.3 (0.93-5.9) | 0.07 |
| CD8^+^ T Naïve | 1.17 | % of total T lymphocytes | 0.54 (0.32-0.89) | 0.016 |
| CD8^+^ T_CM_ | 0.27 | % of total T lymphocytes | 0.52 (0.31-0.87) | 0.012 |
| CD8^+^ T_EM_ | 11.57 | % of total T lymphocytes | 1.7 (1-2.9) | 0.037 |
| CD8^+^ T_EMRA_ | 14.25 | % of total T lymphocytes | 1.8 (1.1-3) | 0.02 |

IHC, immunohistochemistry; HR, hazard ratio; CI, confidence interval; PERLS, CD8^+^ T cell PD-1^+^ to CD4^+^ T cell PD-1^+^ ratio; CM, central memory; EM, effector memory; T_EMRA_, terminally differentiated effector memory T cells expressing CD45RA.

**Supplementary Table 4. Bootstrap-validated hazard ratios (B=200) from multivariable Cox regression models for progression-free survival (PFS).** (A) Model 1: variables selected by univariate analysis (p<0.1 in both PFS and OS analyses), (B) Model 2: variables selected by LASSO regularization based on PFS, (C) final multivariable Cox regression model incorporating variables consistently significant across both models (CD4^+^ T TIGIT^+^ and CD8^+^ T_EMRA_) and clinical covariates. *HR, Hazard raio; CI, confidence interval; OS, overall survival; PERLS, CD8+ T PD-1+ to CD4+ T PD-1+ ratio*

| **(A)** |  |  |  | **(B)** |  |  |
| --- | --- | --- | --- | --- | --- | --- |
| **Model 1. Univariate-selected variables (p<0.1 in PFS and OS)** | | |  | **Model 2. Six variables selected by LASSO (PFS-based)** | | |
| **Variable** | **Bootstrap HR** | **95% CI** |  | **Variable** | **Bootstrap HR** | **95% CI** |
| Stage (IVA vs. IVB) | 0.74 | 0.27 - 1.49 |  | Stage (IVA vs. IVB) | 0.91 | 0.46 - 1.96 |
| Treatment | 0.99 | 0.44 - 2.22 |  | Treatment | 1.35 | 0.63 - 3.48 |
| IL-6 | 2.05 | 0.71 - 9.19 |  | Treg PD1^+^ | 0.55 | 0.27 - 1.1 |
| PERLS | 0.50 | 0.16 - 1.36 |  | CD4^+^ T TIGIT^+^ | 3.12 | 1.44 - 8.09 |
| Treg PD1^+^ | 0.52 | 0.24 - 1.19 |  | CD8^+^ T CTLA4^+^ | 0.72 | 0.32 - 1.49 |
| CD4^+^T TIGIT^+^ | 2.78 | 1.32 - 6.19 |  | CD8^+^ T Naïve | 0.60 | 0.23 - 1.35 |
| CD4^+^ T_EM_ | 2.56 | 0.61 - 32.04 |  | CD8^+^ T_CM_ | 0.64 | 0.27 - 1.38 |
| CD8^+^ T_EMRA_ | 2.21 | 1.29 - 4.27 |  | CD8^+^ T_EMRA_ | 2.22 | 1.29 - 4.5 |

| **(C)** |  |  |
| --- | --- | --- |
| **Variable** | **Bootstrap HR** | **95% CI** |
| Age (≥70) | 1.56 | 0.83 - 2.98 |
| Stage (IVA vs. IVB) | 0.83 | 0.48 - 1.54 |
| Treatment | 1.43 | 0.7 - 3.58 |
| CD4^+^T TIGIT^+^ | 3.86 | 2.3 - 7.99 |
| CD8^+^ T_EMRA_ | 2.13 | 1.26 - 3.85 |

**Supplementary Figure 5. Progression-free survival and immune mediator profiles according to circulating CD4⁺ T TIGIT⁺ levels in patients treated with ICI.** Progression-free survival (PFS) curves for patients with CD4⁺ TIGIT-low and TIGIT-high CD4^+^ T cell groups in the (A) ICI monotherapy and (B) ICI plus chemotherapy cohorts. (C) Comparison of plasma cytokine and cytolytic molecule levels between patients with low and high CD4⁺ T TIGIT groups.

**Supplementary Figure 6. Patient-level correlation between circulating CD4⁺ T cell subsets and TIGIT expression frequency.** Spearman correlation analysis between the frequency of TIGIT-expressing CD4+ T cells and the frequencies of CD4+ T cell differentiation subsets (A: T naïve, B: T_CM_, C: T_EM_, D: T_EMRA_) , Treg (E: CD4+CD25hiCD127lo) and CD8+ T_EMRA_ (F) in individual patients (n=74). Each dot represents one patient. Frequencies are expressed as percentage of total T lymphocytes. Red regression lines indicate statistically significant correlations (p<0.05); grey lines indicate non-significant correlations. Shaded areas represent 95% confidence intervals. *T_CM_, central memory T cell; T_EM_, effector memory T cell; T_EMRA_, terminally differentiated effector memory T cell re-expressing CD45RA; Treg, regulatory T cell.*
